# Supplementary material for: Sequence Analysis of the Complete Mitochondrial Genome of a Medicinal Plant, Vitex rotundifolia Linnaeus f. (Lamiales: Lamiaceae)
Source: Genes (Basel). 2022 May 8;13(5):839. doi: 10.3390/genes13050839 (PMC9140901; doi:10.3390/genes13050839)
Supplement: Supplementary file 1 [file genes-13-00839-s001.zip › genes-1713077-supplementary.pdf]

SUPPLEMENTARY MATERIALS

Sequence analysis of the complete mitochondrial genome of a medicinal plant, *Vitex rotundifolia* Linnaeus f. (Lamiales, Lamiaceae)

Xiaoli Yu1*, Yanjun Wang1, Qingxin Zhang1, Zhonggang Duan1, Wei Li2*

1School of Life Science, Huizhou University, Huizhou 516007, China
2College of Landscape Architecture and Forestry, Qingdao Agricultural University, Qingdao 266109, China
* Corresponding author. School of Life Science, Huizhou University, Huizhou 516007, China. College of Landscape Architecture and Forestry, Qingdao Agricultural University, Qingdao 266109, China.
E-mail address:yuxiaoli@hzu.edu.cn (XLY); Wei Li: lwcsu_caf@163.com (WL).

E-mails:
XLY: yuxiaoli@hzu.edu.cn
YJW: yanjun_wang8204@126.com
QXZ:lazqx@hzu.edu.cn
ZGD: duanzhonggang@163.com
WL:lwcsu_caf@163.com

Table S1. Summary of sequencing reads obtained from the Nanopore platforms.

Bases number	6,621,203,022 bp		
GC content	35.64%		
Reads number	252,868		
Max.	153,918 bp	Min.	105 bp
Mean	26,184 bp		
N50	34994 bp		
N90	16973 bp		

Table S2. Simple Sequence Repeats in the *V. rotundifolia* mitochondrial genome.

Repeats	3	4	5	6	7	8	9	10	11	12	total
A/T	-	-	-	-	-	-	-	9	2	1	12
C/G	-	-	-	-	-	-	-	4	-	-	4
AG/CT	-	-	8	2	-	-	-	-	-	-	10
AT/AT	-	-	5	2	-	-	-	-	-	-	7
AAC/GTT	-	1	-	-	-	-	-	-	-	-	1
AAG/CTT	-	7	1	-	-	-	-	-	-	-	8
AAT/ATT	-	7	-	-	-	-	-	-	-	-	7
ACT/AGT	-	1	-	-	-	-	-	-	-	-	1
AAAG/CTTT	12	-	-	-	-	-	-	-	-	-	12
AAAT/ATTT	2	-	-	-	-	-	-	-	-	-	2
AACG/CGTT	2	-	-	-	-	-	-	-	-	-	2
AAGC/CTTG	3	-	-	-	-	-	-	-	-	-	3
AAGG/CCTT	2	-	-	-	-	-	-	-	-	-	2
AATC/ATTG	2	-	-	-	-	-	-	-	-	-	2
AATG/ATTC	4	-	-	-	-	-	-	-	-	-	4
AATT/AATT	1	-	-	-	-	-	-	-	-	-	1
ACCG/CGGT	1	-	-	-	-	-	-	-	-	-	1
ACTC/AGTG	1	-	-	-	-	-	-	-	-	-	1
ACTG/AGTC	1	-	-	-	-	-	-	-	-	-	1
AGAT/ATCT	1	1	-	-	-	-	-	-	-	-	2
AGCC/CTGG	1	-	-	-	-	-	-	-	-	-	1
AGCG/CGCT	1	-	-	-	-	-	-	-	-	-	1

AGCT/AGCT	1	-	-	-	-	-	-	-	-	-	1
CCGG/CCGG	1	-	-	-	-	-	-	-	-	-	1
AAAAG/CTTTT	1	-	-	-	-	-	-	-	-	-	1
AAATT/AATTT	1	-	-	-	-	-	-	-	-	-	1
AACTT/AAGTT	1	-	-	-	-	-	-	-	-	-	1
AAGAG/CTCTT	1	-	-	-	-	-	-	-	-	-	1
AATAT/ATATT	1	-	-	-	-	-	-	-	-	-	1
AATCT/AGATT	1	-	-	-	-	-	-	-	-	-	1
ACTAG/AGTCT	1	-	-	-	-	-	-	-	-	-	1

Table S3. Tandem repeats in the mitochondrial genome of the *V. rotundifolia*.

accession_number	Indices	Period size(bp)	Copy Number	Consensus Size (bp)	Percent Matches	Percent Indels	Score	Bases number				Entropy
								A	G	C	T	(0–2)
OK563725.1/2	91352-91459	54	2	54	96	0	198	26	26	12	34	1.92
OK563725.1/2	173866-173895	15	2	15	93	0	51	10	16	0	73	1.09
OK563725.1/2	223039-223068	15	2	15	93	0	51	23	20	10	46	1.8
OK563725.1/2	258077-258120	17	2.6	17	78	3	54	18	18	34	29	1.94

Table S4. Chloroplast DNA insertions in the *V. rotundifolia* mitochondrial genome.

Query	Subject	Identity (%)	Alignment Length	Mismatches	Gap openings	Plastome		Mitogenome Sequence		E value	Score
						Start	End	Start	End		
NC_050991.1	V.rotundifolia	98.737	475	5	1	101523	101996	281577	281103	0	843
NC_050991.1	V.rotundifolia	98.737	475	5	1	137454	137927	281103	281577	0	843
NC_050991.1	V.rotundifolia	97.24	471	2	2	85667	86127	366970	366501	0	787
NC_050991.1	V.rotundifolia	97.24	471	2	2	153323	153783	366501	366970	0	787
NC_050991.1	V.rotundifolia	98.206	223	4	0	100977	101199	95110	94888	1.37E-107	390
NC_050991.1	V.rotundifolia	98.206	223	4	0	138251	138473	94888	95110	1.37E-107	390
NC_050991.1	V.rotundifolia	83.61	421	42	13	67398	67811	10835	11235	1.79E-101	370
NC_050991.1	V.rotundifolia	91.732	254	10	4	1086	1329	209025	209277	3.90E-93	342
NC_050991.1	V.rotundifolia	98.851	174	2	0	137981	138154	94080	93907	1.10E-83	311
NC_050991.1	V.rotundifolia	98.851	174	2	0	101296	101469	93907	94080	1.10E-83	311
NC_050991.1	V.rotundifolia	73.957	887	175	40	137279	138142	42657	41804	1.42E-82	307
NC_050991.1	V.rotundifolia	73.957	887	175	40	101308	102171	41804	42657	1.42E-82	307
NC_050991.1	V.rotundifolia	88.018	217	21	1	73731	73947	242170	242381	6.76E-66	252
NC_050991.1	V.rotundifolia	91.16	181	12	1	45893	46073	346226	346402	4.07E-63	243
NC_050991.1	V.rotundifolia	79.109	359	67	5	107375	107727	125508	125152	1.46E-62	241
NC_050991.1	V.rotundifolia	79.109	359	67	5	131723	132075	125152	125508	1.46E-62	241
NC_050991.1	V.rotundifolia	75.055	453	101	11	10895	11341	90669	90223	2.48E-50	200

NC_050991.1	V.rotundifolia	87.791	172	14	3	46657	46828	346420	346584	1.16E-48	195
NC_050991.1	V.rotundifolia	75.378	463	79	22	106647	107093	126217	125774	1.50E-47	191
NC_050991.1	V.rotundifolia	75.378	463	79	22	132357	132803	125774	126217	1.50E-47	191
NC_050991.1	V.rotundifolia	84.524	168	26	0	36711	36878	57808	57975	2.52E-40	167
NC_050991.1	V.rotundifolia	78.03	264	56	2	136989	137252	43031	42770	9.06E-40	165
NC_050991.1	V.rotundifolia	78.03	264	56	2	102198	102461	42770	43031	9.06E-40	165
NC_050991.1	V.rotundifolia	92.174	115	8	1	31272	31385	345706	345592	1.17E-38	161
NC_050991.1	V.rotundifolia	75.521	192	43	4	106009	106197	127036	126846	1.56E-17	91.6
NC_050991.1	V.rotundifolia	75.521	192	43	4	133253	133441	126846	127036	1.56E-17	91.6

Table S5. The list of accession numbers of the mitogenome sequences used in the phylogenetic analyses.

No.	Taxon	Family	Accession Number
1	*Vitex rotundifolia*	Lamiaceae	OK563725/6.1
2	*Scutellaria tsinyunensis*	Lamiaceae	MW553042.1
3	*Ajuga reptans*	Lamiaceae	NC_023103.1
4	*Rotheca serrata*	Lamiaceae	NC_049064.1
5	*Salvia miltiorrhiza*	Lamiaceae	NC_023209.1
6	*Boea hygrometrica*	Gesneriaceae	NC_016741.1
7	*Utricularia reniformis*	Lentibulariaceae	NC_034982.1
8	*Hesperelaea palmeri*	Oleaceae	NC_031323.1
9	*Castilleja paramensis*	Orobanchaceae	NC_031806.1
10	*Erythranthe lutea*	Phrymaceae	NC_018041.1
11	*Nicotiana tabacum*	Solanaceae	NC_035963.1
12	*Solanum lycopersicum*	Solanaceae	NC_006581.1

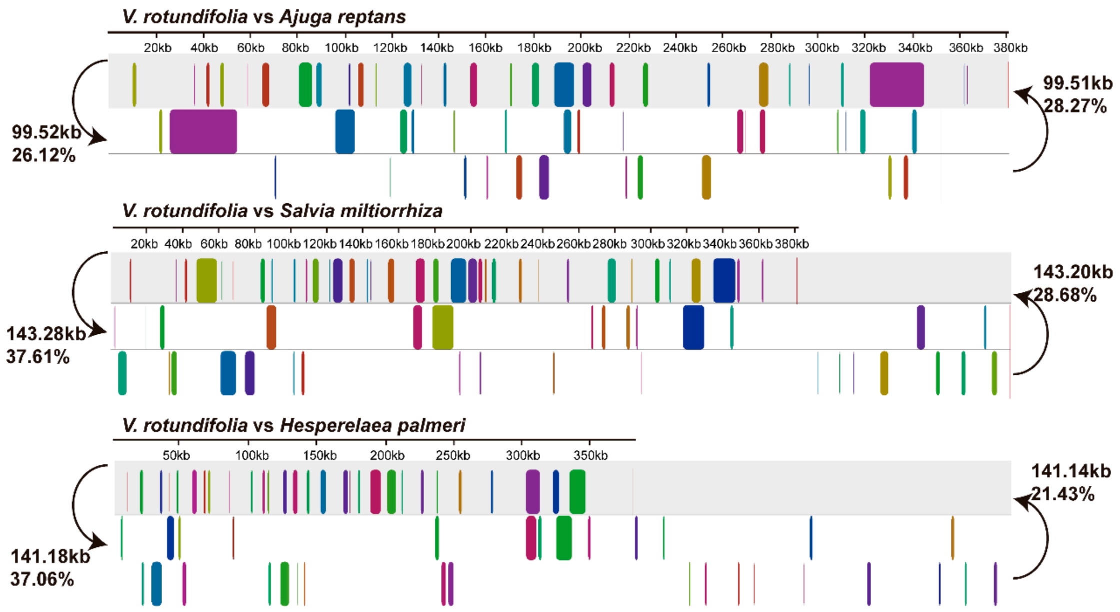

Figure S1. Synteny blocks and shared mtDNA of the *V. rotundifolia* with other 3 species of the Lamiales based on MAUVE alignments. Left arrows showed the shared amount of mtDNA, in kb and percentage, of the *V. rotundifolia* with the other one species, and right arrows showed the reciprocal values.

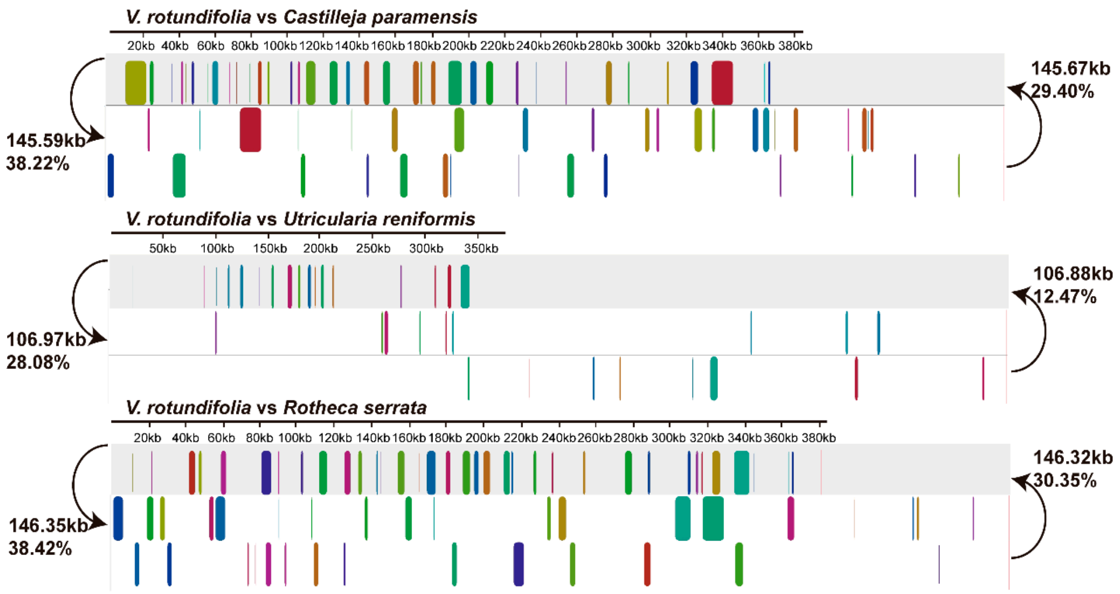

Figure S2. Synteny blocks and shared mtDNA of the *V. rotundifolia* with other 3 species of the Lamiales based on MAUVE alignments. Left arrows showed the shared amount of mtDNA, in kb and percentage, of the *V. rotundifolia* with the other one species, and right arrows showed the reciprocal values.

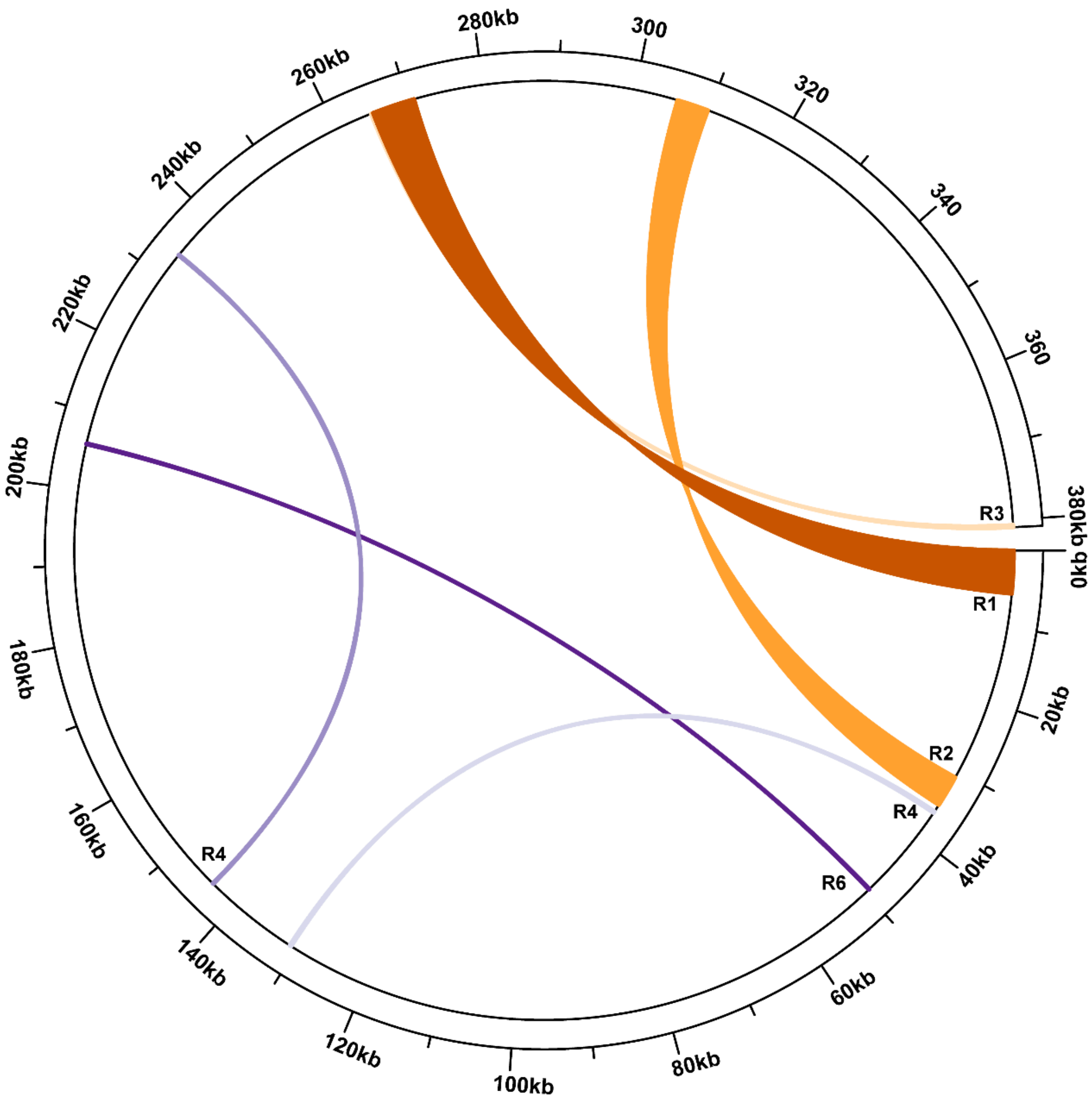

Figure S3. Locations of six direct repeats in the mitochondrial genome of *Vitex rotundifolia*.

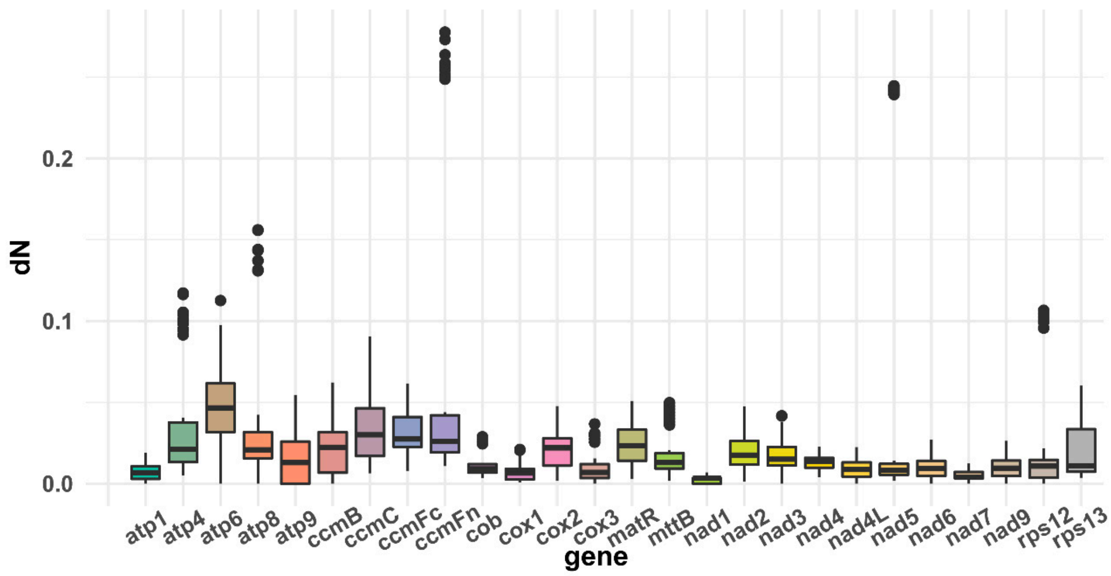

Figure S4. Boxplots of pairwise dN values for the mitochondrial genes among the ten Lamiales plants.

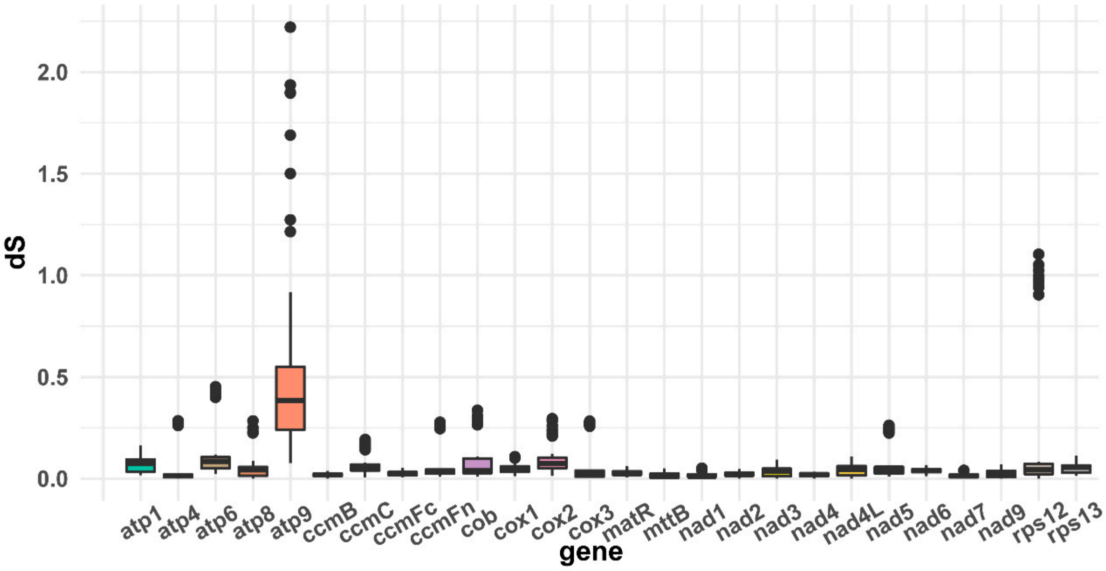

Figure S5 Boxplots of pairwise dS values for the mitochondrial genes among the ten Lamiales plants.
